# The Week's Work

**Published:** 1910-11-26

**Authors:** 


### The Week's Work.

### REFORM OF CHARITIES' HELP IN AUSTRALIA.

Overlapping of charities is again attracting attention in several States of the Australian Commonwealth. The subject was started at the annual meeting of the Melbourne Charity Organisation Society. The Victorian State Treasurer was interviewed and stated that he was preparing a Charities Bill for the present Session of Parliament, and that it would be "a rigidly practical measure." The Queensland Government is already considering the matter, and, it is reported, has virtually decided to donate to the hospitals the money now received from the tax on totalisators. For the last three years the amounts received from this source have been £11,000, £12,400, and £12,800, in round numbers. The Government will also place a tax on all tickets for amusements, and arrangements will be made to collect a small sum weekly from persons in receipt of a certain wage, as is already voluntarily done in many Government departments and places of business. A proportion of the income tax is also mentioned.

### A CHILDREN'S HOSPITAL FOR LEEDS.

The *Leeds Hospital Magazine* contains a protest against the proposal of two local medical men who have written to the *Yorkshire Post* advocating the establishment of a new and separate Children's Hospital in that town as a memorial to King Edward VII. The journal points out that ample accommodation already exists at the Infirmary and the Hospital for Women and Children. The first-named institution is greatly in need of financial help, not only to repair the damage caused by the recent fire which destroyed the children's portion of one of the hospital blocks, but also for the provision of new theatres, new heating-boilers, new kitchen-ranges, and other pressing wants. An appeal to the public for £100,000 will probably be launched before long. Again, the New Maternity Hospital is badly in want of £5,000 to set it on a proper footing. In the opinion of the journal it would be far better to direct attention to the needs of the existing institutions than to indulge a passion for bricks and mortar by the erection of new buildings for hospitals.

### DISINFECTION OF FLOORS IN L.C.C. SCHOOLS.

The Public Health and Housing Committee of the Poplar Borough Council has recently, with the consent of the London County Council, inaugurated an experimental spraying and cleansing of the floors of six of the County Council schools situated in the borough of Poplar, with electrolytic disinfecting fluid. For the purposes of the experiment the infants' departments only are being treated in this way at present. The sawdust used for scattering over the floors is well soaked in the electrolytic fluid, and the floor is sprinkled with the fluid by means of an ordinary galvanised iron watering-can with a large rose on the spout. The soaked sawdust is then scattered over the floor, after which the latter is swept. When it is necessary to wash the floors, the fluid is mixed with the water used for that purpose. Up to August 31, 596 gallons of the fluid have been supplied to the schools. The experiment is being watched closely to see whether the attendances in the schools selected are better than those of others. The general public is also supplied with the fluid for domestic purposes. Seven depôts exist for its distribution, and up to August of this year some 16,572 gallons have been given out in this way. In addition another 16,906 gallons have been used in the baths, workhouses, offices, shelters, works, training schools, and public halls belonging to the borough council. The total cost of materials to produce the 33,478 gallons used up to August of this year was £62 7s. 6d., while the cost of bottles, corks, and labels was £24 10s. 6d.

### HOSPITAL LEGISLATION IN VICTORIA.

Private hospitals have increased largely in number in Victoria of late years. The licensing of these has been in the hands of the municipalities, but has been allowed to become a dead letter. A new Bill is being prepared which will take this power from the Town Councils and vest it in the Government. Not only must private hospitals be licensed, but subject to Government supervision and inspection. The Inspector of Charities holds that the establishment of intermediate hospitals where people of limited means could get medical attention and nursing at moderate rates is a necessity. Such hospitals would relieve the public institutions, now largely used by those of small means. The Inspector thinks that the medical profession is the chief obstacle to this reform, as the medical societies insist that the question of fees must be left to doctor and patient; thus the latter never know, until the bills are presented, what the fees will amount to, but these hospitals were where the scale of medical and nursing fees was fixed, patients would prefer them to the public institutions. Many would be glad to pay, but do not care to be ruined. It is thought that, failing private enterprise, the Government should establish these hospitals, and devote the profits to the charities.

### A MODEL HOSPITAL REPORT.

We have just received from Dr. G. van Eysselsteyn, Medical Superintendent of the Groningen General Hospital, a copy of the memorial report of the institution over which he presides. We wish we could enable every hospital secretary to see this interesting and remarkable volume, which is really one of the finest reports on hospital construction and management we have as yet had the pleasure of reading. It is well printed, beautifully illustrated, and the reading matter is instructive in the highest degree. The report is, in fact, a monograph on hospital construction which should be known to everyone who takes a technical interest in the question. It is published by Messrs. Noordhoff, of Groningen, and we hope to review it *in extenso* in an early number of The Hospital.

### HOSPITAL ADVERTISEMENT—THE WRONG WAY.

Lately various paragraphs have gone the round in the daily and weekly Press concerning what is styled " one of the most remarkable operations ever attempted," and with this epoch-making discovery of the illimitable possibilities of surgery the name of a well-known London hospital has been prominently associated. So far as can be gathered from the descriptions, the operation in question appears to have been one of the simple plastic attempts to restore continuity of tissue. Its principle, and, for that, its application, are as old as Erasistratus, and will be familiar enough to our professional readers who are acquainted with what is known as the Indian method of rhinoplasty. There is, therefore, nothing specially remarkable in it, and dozens of operations much more wonderful and much more worthy of comment, if lay comment in such matters is at all desirable (which we venture very much to doubt), are daily performed in every operating room in this country. It is possible that the surgeon who so frankly discussed his case with a lay reporter thought that he was doing the institution to which he belongs some good by procuring it a gratuitous advertisement. If so, the sooner he is disillusioned by his colleagues and his governors the better. No hospital can endeavour with justice to win subscriptions by such methods of self-advertisement. An individual operation such as this demands no exposition in the public Press; the discussion of its merits has already had a fair amount of space in the columns of the professional Press. Such wide publicity as has been given to this isolated case is harmful in that it tends to exalt the institution in the public eye above the numerous other hospitals, where equally, and more, meritorious work is daily done without parade and ostentation. Where a new method of treatment demands additional outlay and extra expense it is necessary that it should be explained, in a regular manner, by the authorities of the hospital to the public at large. But medical men, no less than hospital workers, will agree with us that scrappy paragraphs of the nature we have alluded to do more harm than good. They are not only in bad taste, from a professional point of view, but they tend by implication to reflect unjustifiably on other institutions where similar advertising tactics are rigorously eschewed by the staff.

### A CHILDREN'S FUND FOR MIDDLESEX.

Prince Alexander of Teck has started a Children's Collection Fund in connection with " The Prince Francis of Teck Memorial Fund," on behalf of the Middlesex Hospital. Books of numbered coupons—each coupon being in itself a receipt for one shilling—are being issued to children who are anxious, with the consent of their parents, to collect for the memorial. Application for permission to become a collector should be made to his Serene Highness at the Middlesex Hospital, Mortimer Street, W., when a book of coupons will be forwarded by return of post. This scheme provides children of every age and of every phase of life with an opportunity of showing their sympathy with the sufferings of the many little ones who are cared for in the three children's wards at the hospital. It would indeed be a splendid thing if the result of their efforts were to raise a sum sufficient to meet that portion of the annual deficit in the hospital's income which is incurred in connection with the maintenance of those children's wards. Offers of help will be many, especially when it is known that the first application for a children's collection book came from her Royal Highness Princess Mary, who has already enlisted many helpers in the good cause.

### THIS WEEK'S DRUG MARKET.

A steady business continues to be done in those drugs which are usually in request at this season of the year, but notwithstanding the cold spell the demand for cod-liver oil is by no means large, and at present prices show no tendency to advance. It is not probable, however, that the value of this article will recede to any appreciable extent, and there seems no good reason to postpone purchases. The expected rise in the cost of glycerine may be announced at any moment, and it would perhaps be wise to buy in sufficient quantity to last through the winter. After the sharp advance in the price of ipecacuanha last week, business in the drug has been on a small scale, but it seems likely that the present comparatively high quotations will be maintained, at any rate for the present. Opium appears to be tending somewhat dearer. Ergot has advanced in price again, and still higher values are probable. Castor oil is slightly dearer. Potassium bromide and other bromine salts are quoted a little higher, and makers still decline to book contracts for future delivery, which may mean a further advance before very long. Hydrobromic acid is slightly higher in price. At the recent drug auctions in Mincing Lane extreme prices were paid for Siam gum benzoin, which is scarce and in good request. Senna sold at prices which were a trifle below those previously paid, and Japanese refined camphor was slightly lower. There seems every prospect of good business in the drug market for some months, but it is feared that the unsettling influence of the General Election will be felt in this as in other markets.

